# Non-Coding RNAs Delivery by Small Extracellular Vesicles and Their Applications in Ovarian Cancer

**DOI:** 10.3389/fbioe.2022.876151

**Published:** 2022-05-19

**Authors:** Mu Liu, Xiaofang Zhou, Jie Tang

**Affiliations:** ^1^ Department of Gynecologic Oncology, Hunan Cancer Hospital, The Affiliated Cancer Hospital of Xiangya School of Medicine, Central South University, Changsha, China; ^2^ Department of Gynecologic Oncology, Hunan Gynecologic Cancer Research Center, Hunan Cancer Hospital, The Affiliated Cancer Hospital of Xiangya School of Medicine, Central South University, Changsha, China

**Keywords:** ovarian cancer, small extracellular vesicles, non-coding RNA, biomarker, therapy, drug delivery

## Abstract

Ovarian cancer (OC) is the most fatal gynecological malignancy because of its early asymptomatic nature and acquired resistance to chemotherapy. Small extracellular vesicles (sEVs) are a heterogeneous group of biological vesicles with a diameter <200 nm released by cells under physiological or pathological conditions. sEVs-derived non-coding RNAs (ncRNAs) are the essential effectors in the biological environment. sEVs-ncRNAs have critical roles in tumor progression via regulating mRNA expression of target cells to affect cell signaling. In addition, the status of parental cells can be disclosed via analyzing the composition of sEVs-ncRNAs, and their “cargoes” with specific changes can be used as key biomarkers for the diagnosis and prognosis of OC. Accumulating evidence has demonstrated that sEVs-ncRNAs are involved in multiple key processes that mediate the development of metastasis and chemotherapeutic resistance in OC: epithelial–mesenchymal transition; tumorigenicity of mesenchymal stem cells; immune evasion; angiogenesis. The nanomedicine delivery system based on engineering sEVs is expected to be a novel therapeutic strategy for OC. Insights into the biological roles of sEVs-ncRNAs in the invasion, metastasis, immune regulation, and chemoresistance of OC will contribute to discovery of novel biomarkers and molecular targets for early detection and innovative therapy. In this review, we highlight recent advances and applications of sEVs-ncRNAs in OC diagnosis and treatment. We also outline current challenges and knowledge gaps.

## Introduction

Ovarian cancer (OC) is the seventh most common cancer in women with a 5-year survival of <50% (Siegel et al., 2021). Often, OC is referred to as the “silent killer”, and its pathological and molecular features are highly heterogeneous (Torre et al., 2018). Over 70% of OC cases are diagnosed at stage III or IV with widespread abdominal metastasis. The 5-year survival of patients with stage-I OC can exceed 90%. The 5-year survival of patients with stage-III–IV disease after aggressive debulking surgery and platinum-based chemotherapy is only 30% (Matulonis et al., 2016; Kuroki and Guntupalli, 2020). High-grade serous ovarian cancer (HGSOC) accounts for 70–80% of OC deaths, because most of them relapse and, ultimately, develop resistance to chemotherapy (Yang et al., 2011). HGSOC is characterized by ubiquitous TP53 mutation (96%), significant focal DNA copy number aberrations (e.g., CCNE1, MYC, MECOM, RB1, and NF1), and homologous recombination deficiency (HRD) in about half of cases (Network Cancer Genome Atlas Research, 2011). In addition, approximately 13% of HGSOC cases can be attributed to germline breast cancer gene 1 (gBRCA1) or gBRCA2 mutation (Pal et al., 2005).

The high mortality of OC is associated with the difficulty of obtaining an early diagnosis and chemoresistance-related relapse. Due to the limited expression of CA125 in early-stage OC and its correlation with non-malignant diseases (e.g., endometriosis and pelvic *tuberculosis*), its positive predictive value is only 40% (Menon et al., 2009; Menon et al., 2021). An effective and accurate screening strategy for OC is not available. The first-line maintenance therapy based on poly-ADP ribose polymerase inhibitor (PARPi) has become an indispensable part of OC clinical management. Regardless of gBRCA mutations or HRD status, PARPi can prolong the median progression-free survival of patients with recurrent OC to some extent (Mirza et al., 2016; Franzese et al., 2019). Unfortunately, most OC patients with platinum-resistant develop resistance to PARPi eventually. Consequently, there is an urgent need to: 1) further clarify the mechanisms of OC with widespread metastasis and chemoresistance; 2) develop novel and reliable biomarkers and molecular targets for an early diagnosis and treatment of OC.

Small extracellular vesicles (sEVs) are submicron-sized vesicles derived from cell endosomes. They have become an alternative source of biomarkers for various diseases (Wong et al., 2019; Guo et al., 2020; Alharbi et al., 2021). sEVs are found in multiple biological fluids (e.g., blood, saliva, urine, and cerebrospinal fluid) and enrich RNA species that represent specific biotypes of source cells, especially non-coding RNAs (ncRNAs) (Möller and Lobb, 2020). The latter are RNAs that do not encode specific proteins but instead act as essential regulators of physiological processes in developmental and disease environments. It has been estimated that ncRNAs regulate the translation of ∼60% of protein-coding genes (Anastasiadou et al., 2018). Increasing evidence suggests that sEVs participate closely in the communication between cancer cells and the tumor microenvironment (TME) by selectively delivering ncRNA “cargo” to alter the genes expression and phenotypic responses of recipient cells (Hoshino et al., 2015; O’Brien et al., 2020; Grunberg et al., 2021). As pathological effectors, sEVs-ncRNAs have critical roles in a variety of cancer hallmarks in OC: oncogenic reprogramming (Kanlikilicer et al., 2016); tumor stem cells maintenance (Cao et al., 2021), DNA damage repair (Chen et al., 2021), and chemotherapy resistance (Alharbi et al., 2021). Owing to their natural origin, sEVs possess excellent biocompatibility, low immunogenicity, enhanced stability, and low toxicity. sEVs have the potential to be novel nanocarriers for drugs compared with traditional nanoscale drug carriers (e.g., liposomes and synthetic nanoparticles) (Zhang et al., 2020d; Herrmann et al., 2021; Jang et al., 2021). Given these biochemical advantages, elucidating the relationship between OC and delivery of sEVs-ncRNAs may aid the development of biomarkers for an early diagnosis and the establishment of novel molecular-targeted therapies.

Due to research priorities in recent years, we will mainly focus on a subtype of sEVs named “exosomes”. However, most experimental methods do not include a sufficiently detailed description of the exosome isolation process to demonstrate their intracellular origins and biogenesis, which may contain heterogeneous EVs populations of different biological origins. Therefore, sEVs are employed to designate EVs <200 nm in diameter, and the separate term of exosome will be employed only in specific descriptions or original publications.

In this review, we summarize the latest development of sEVs-derived ncRNAs as OC biomarkers and therapeutic targets in diagnosis, surveillance, and treatment. We highlight the roles of ncRNAs delivery via sEVs in the pathogenesis and progression of OC, the potential translational applications of engineering sEVs in OC treatment, as well as the challenges and possible knowledge gaps that will be faced.

## Biogenesis and “Packaging” of sEVs

“EV” is a generic term for particles with no functional nucleus naturally released from all cells in eukaryotes and prokaryotes. This definition is endorsed by the International Society for Extracellular Vesicles (ISEV) (Lotvall et al., 2014; Thery et al., 2018). The intercellular transfer of EVs has garnered extensive attention as a novel third mechanism of intercellular communication (in addition to direct cell-to-cell contact and secretion of growth factors and cytokines) (Andaloussi et al., 2013; Tkach and Thery, 2016; Raposo and Stahl, 2019). Activated vesicles were first discovered in 1983 during the *in vitro* culture of sheep reticulocytes, and were named “exosomes” by Johnstone et al. (1987). Exosomes were originally thought to be cellular “debris”: a way for cells to excrete waste. In recent years, the EV-research community has reached a consensus to categorize EVs broadly into three main types depending on their biogenesis mechanisms (Colombo et al., 2014). Apoptotic bodies are the largest EVs (diameter = 800–5,000 nm) and are formed from budding cells undergoing apoptosis. Mid-sized EVs (e.g., microvesicles, microparticles, and ectosomes) are produced by direct outward budding of the plasma membrane, and range in diameter from 100 to 1,000 nm. Exosomes are the smallest EVs (40–160 nm in diameter). They are exocytosed intraluminal vesicles (ILVs) released by the fusion of multivesicular bodies (MVBs) with the cell membrane (Kalluri and LeBleu, 2020).

**FIGURE 1:**
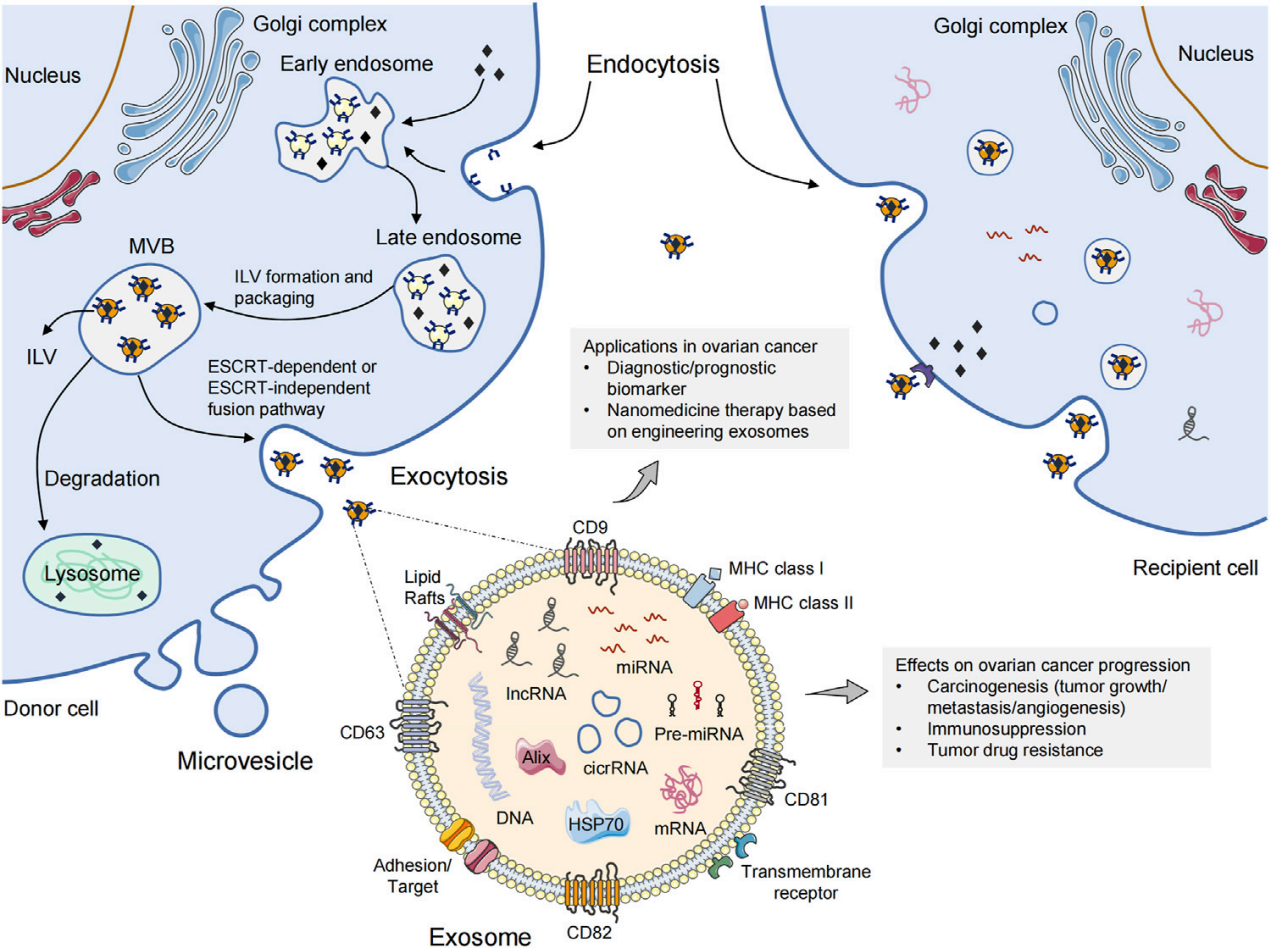
The biogenesis and cellular-uptake of extracellular vesicles. Microvesicles are released directly from the plasma membrane *via* outward budding. The biogenesis of exosomes follows the endocytic-exocytic pathway, inward budding from the plasma membrane to form early endosomes. Late endosomal membrane invagination results in the loading of cytosolic proteins/RNAs, which further forms the exosomal precursor–intraluminal vesicles (ILVs) in multivesicular bodies (MVBs). Exosomes are then secreted into the extracellular space after fusion with the plasma membrane via an ESCRT-dependent or ESCRT-independent pathway. Exosomes can be taken up by recipient cells through endocytosis, direct fusion, or binding to surface proteins, causing the transfer of oncogenic cargoes, and changing the biological behaviors of recipient cells. A multitude of non-coding RNA species (e.g., miRNAs, circRNAs, lncRNAs) are contained in exosome. The roles of exosomes in ovarian cancer are also shown. ESCRT, endosomal sorting complex required for transport; MHC, major histocompatibility complex; miRNA, microRNA; circRNA, circular RNA; lncRNA, long non-coding RNA.

The endosomal sorting complex required for transport (ESCRT) pathway is the most well-known pathway in exosomal biogenesis and cargo classification (Maas et al., 2017; van Niel et al., 2018). This pathway mainly involves four core complexes (ESCRT 0, I, II, and III) that identify ubiquitinated membrane proteins and induce membrane budding, which leads to the formation of ILVs and MVBs (Morita et al., 2007). Briefly, ESCRT-0 recognizes and sorts ubiquitinated endosomal membrane proteins. Further binding to ESCRT-I and ESCRT-II causes inward budding of the endosomal membrane to allow capture of different cargoes (Katzmann et al., 2001). Then, the ESCRT-III complex undergoes deubiquitination and cleaves the budding vesicles (Chiaruttini et al., 2015). This process is coordinated by multiple ancillary proteins in the ESCRT pathway, including ALG2-interacting protein X (ALIX), tumor susceptibility gene 101 (TSG101), and L domain-containing proteins (Christ et al., 2017). These budding vesicles form early endosomes and eventually mature into late endosomes and MVBs. Most MVBs are degraded via fusion with lysosomes. Only a few MVBs containing CD63 and lysosomal-associated membrane protein 1 (LAMP1) fuse with the plasma membrane to release exosomes exocytotically, which is facilitated by Rab guanosine triphosphatase (GTPase) 27a and 27b (Ostrowski et al., 2010). In addition, cells can also generate ILVs and MVBs independent of the ESCRT pathway through lipids, ceramides, tetraspanins, or heat-shock proteins (HSPs) (Shao et al., 2018). These specific membrane transport and fusion processes lead to the presence of typically marker proteins in exosomes. Such marker proteins for exosome recognition include CD9, CD81, CD63, TSG101, ALIX, and HSP70 (Pegtel and Gould, 2019). Exosomes also contain unique cell function-related proteins. For example, major histocompatibility complex (MHC) class-I or class-II molecules are abundant in exosomes derived from antigen-presenting cells (APCs) (Andre et al., 2002). Exosomes secreted by epithelial tumor cells carry epithelial cell adhesion molecule (EpCAM) (Takao et al., 2018). Exosomes released from breast cancer (Ciravolo et al., 2012) and gastric cancer (Baran et al., 2010) cells express human epidermal receptor family proteins. Importantly, the distinct nucleic-acid, protein, and lipid contents of exosomes are not merely a reflection of donor cells. Studies have shown that the expression profile of exosomal cargoes is different from that of the original cells to some extent, which indicates a highly controlled sorting process (Zaborowski et al., 2015). The interaction between exosomes and recipient cells is dependent upon specific glycans, lipids, proteins, and total negative charge on their membrane surface. The cellular-uptake pathways of exosomes mainly include receptor-mediated endocytosis, clathrin interaction, micropinocytosis, direct fusion, and interaction between “lipid rafts”. The biogenesis and cellular-uptake mechanisms of EVs are presented in Figure 1.

Assigning EVs to specific biogenerative pathways is extraordinarily difficult unless they are captured via the real-time imaging system during release (Gould and Raposo, 2013). Therefore, if reliable markers of subcellular origin cannot be established, ISEV recommends using size-based classification terms for EV subtypes, such as “sEVs” and “medium/large EVs” (Thery et al., 2018). Additionally, technical problems hinder the extraction and purification of sEVs to some extent (Tian et al., 2020). Protein aggregates, cell debris, viruses, and liposomes must be removed to purify sEVs completely. There are five main methods for sEVs isolation: differential ultracentrifugation (Thery et al., 2006); sucrose and iodixanol density ultracentrifugation (Kowal et al., 2016); size-exclusion chromatography (Hong et al., 2016); polyethylene-glycol precipitation (Weng et al., 2016); and immunoaffinity capture (Cai et al., 2018). In the future, in-depth understanding of the biogenesis and packaging mechanisms of sEVs may have vital roles in optimization of isolation methods.

## Functional Delivery of ncRNAs by sEVs

Non-coding nucleic acid sequences (which do not encode proteins) account for about 98% of the human genome. In general, the biological complexity of organisms is positively correlated with these non-protein-coding sequences, which are widely transcribed into a large number of ncRNAs (Esteller, 2011; Slack and Chinnaiyan, 2019). During the biogenesis of sEVs, cellular-vesiculation mechanisms package multiple ncRNAs into different EVs subclasses to form a substantial pool of extracellular ncRNAs (Fabbiano et al., 2020). With the development of high-throughput sequencing, a multitude of ncRNA species contained in sEVs have been identified, including some highly conserved (e.g., microRNAs [miRNAs], circular RNAs [circRNAs]), as well as others lacking conservation across species (e.g., long non-coding RNAs [lncRNAs]) (Pegtel and Gould, 2019). Agilent Bioanalyzer 2,100 analysis shows that sEVs have a higher content of small RNAs (miRNA) than large EVs, and no prominent ribosomal RNA peaks (Crescitelli et al., 2020).

Small size, high abundance, and targeted localization aid sEVs-mediated intercellular transport of specific ncRNAs (Das et al., 2019). The lipid bilayer of sEVs can protect ncRNAs from RNase degradation during transport (Min et al., 2019). Once sEVs-ncRNAs escape the degradation pathway and are delivered to recipient cells, they can elicit the corresponding functional response. There may be a functional correlation between sEVs biogenesis and miRNA-regulated mRNA silencing (Murphy et al., 2019; Garcia-Martin et al., 2022). Gibbings et al. (2009) have found that GW182, an important component of miRNA-containing RNA-induced silencing complex (miRISC), is enriched in monocyte-derived exosomes. Moreover, depletion of some ESCRT-related components (ALIX, vacuolar protein-sorting-associated protein 36 [VPS36], and hepatocyte growth factor-regulated tyrosine kinase substrate [HRS]) reduce miRNA activity. Hence, interference with the integrity of ESCRT limits miRNA function, possibly via altering MVBs uptake of GW182. Mittelbrunn et al. (2011) have discovered a novel mechanism of cellular communication: CD63 ^+^ exosomes transfer miRNAs unidirectionally between cells using immunological synapses. They also have found that T cells-, B cells-, and dendritic cells-derived exosomes contained miRNA repertoires that are different from their parent cells. Targeting neutral sphingomyelinase-2 can inhibit exosomes production and destroy the transfer of miRNAs to APCs. Other EV-modification signals (e.g., ubiquitination, uridylation, phosphorylation, and sumoylation) also affect the splicing and translation of RNA, and miRNA biogenesis (Mukherjee et al., 2016; Montaldo et al., 2021). The effect of these mechanisms on RNA packaging of sEVs depends largely on particular RNA species, and how cells regulate RNA-cargo loading of sEVs is not known. Thus, a deeper understanding of the complex network of sEVs-ncRNAs synergies may provide a unique opportunity to design better diagnostic methods and therapeutic interventions for OC.

## sEVs-ncRNAs in Oncogenic Alterations of OC

**FIGURE 2:**
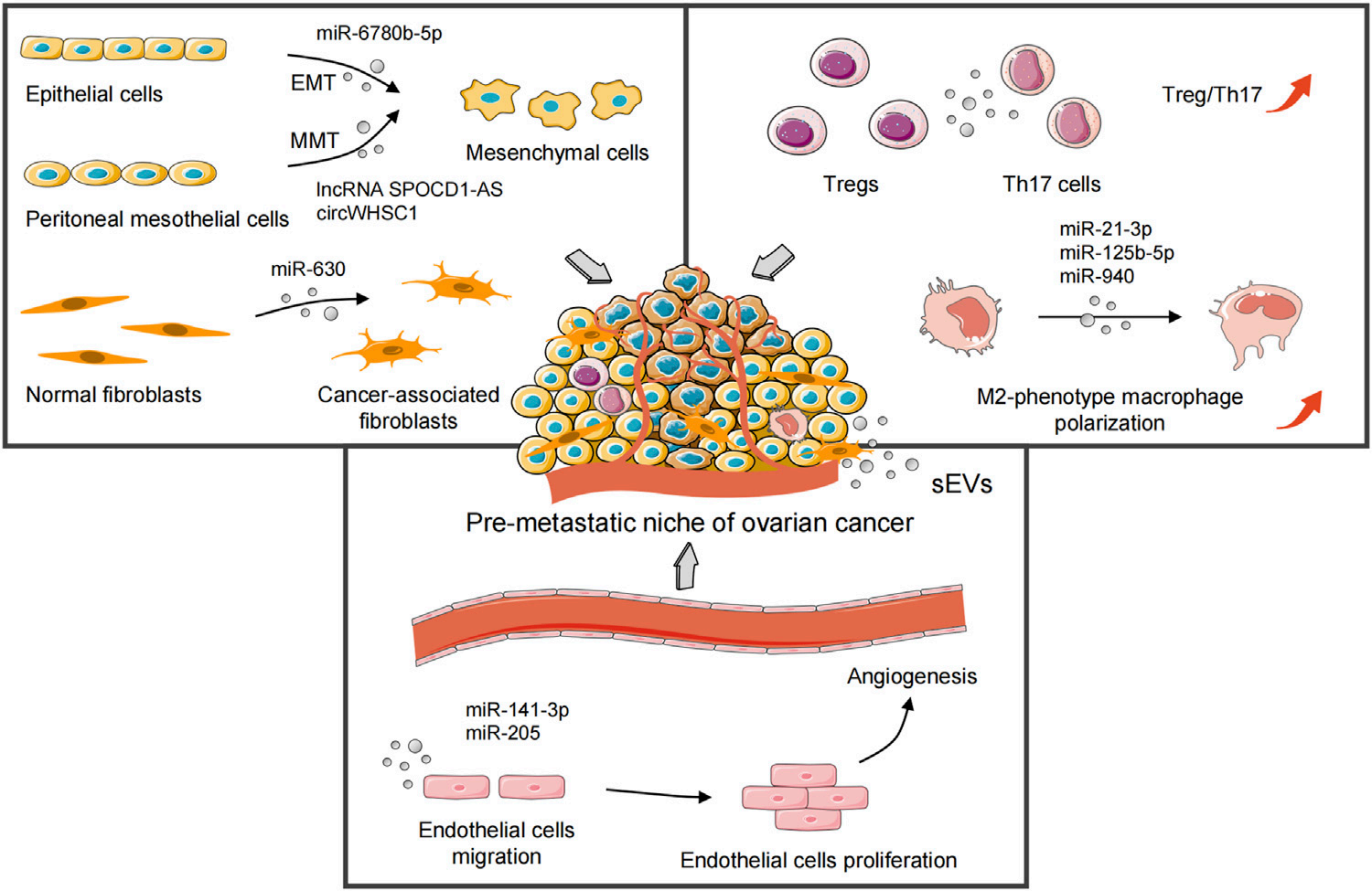
sEVs-derived oncogenic ncRNAs mediate formation of the pre-metastatic niche of ovarian cancer by local stromal remodeling, immunosuppression and evasion, and angiogenesis. sEVs, small extracellular vesicles; EMT, epithelial–mesenchymal transition; MMT, mesothelial–mesenchymal transition; Tregs, regulatory T cells; Th17 cells, T helper 17 cells.

In general, OC is characterized by widespread metastatic growth in the peritoneal cavity and accumulation of malignant ascites (Feng W. et al., 2019). Studies have shown an abundance of exosomes in the ascites of OC patients. They promote OC progression via clearing the interstitial barrier and remodeling the peritoneal environment (Graves et al., 2004; Yamamoto et al., 2018). Oncogenic ncRNAs can be transmitted horizontally to recipient cells in local and distant microenvironments via sEVs. Then, sEVs mediate formation of the pre-metastatic niche of OC by local stromal remodeling (Wang C. et al., 2021), immunosuppression and evasion (Zhou et al., 2018), and angiogenesis (He et al., 2019) (Figure 2). This sEVs-mediated pre-metastatic microenvironment is coordinated through reciprocal interplay with multiple components in the TME, including the extracellular matrix (chemokines and proinflammatory cytokines) and stromal cells (peritoneal mesothelial cells [PMCs], fibroblasts, macrophages, and endothelial cells) (Hoshino et al., 2015). Furthermore, the release of cancer-derived and TME-derived sEVs is increased under various types of microenvironmental crosstalk in inflammation (Li G. et al., 2021), oxygen tension (Dorayappan et al., 2018; Lian et al., 2020), and the therapeutic pressures from irradiation (Zhang F. et al., 2021) or chemotherapy (Zang et al., 2021), and the biomolecular properties of sEVs-ncRNAs are changed accordingly. Table 1 summarizes the relevant studies of sEVs-ncRNAs in OC carcinogenesis.

**TABLE 1:** Summary of sEVs-ncRNAs involved in OC carcinogenesis.

sEVs-ncRNAs	Source	Recipient Cells	Roles/Mechanisms	References
miR-6780b-5p	Ascites	OC cells	Induces epithelial–mesenchymal transition of OC cells	Cai et al. (2021)
miR-330-3p	Plasma cells	OC cells	Promotes core epithelial-mesenchymal transition programs	Yang et al. (2021b)
lncRNA SPOCD1-AS	OC cells	Peritoneal mesothelial cells	Remodels mesothelial cells via interacting with G3BP1	Wang et al. (2021a)
circWHSC1	OC cells	Peritoneal mesothelial cells	Sponges miR-145 and miR-1182 to promote proliferation and invasion of OC cells	Zong et al. (2019)
circPUM1	OC cells	Peritoneal mesothelial cells	Sponges miR-615-5p and miR-6753-5p to inhibit apoptosis via up-regulating NF-κB and MMP2	Guan et al. (2019)
miR-630	OC cells	Normal fibroblasts	Facilitates CAFs activation	Cui et al. (2021)
miR-221-3p	M2-phenotype macrophages	OC cells	Promotes proliferation and G1/S transition of OC cells	Li and Tang, (2020)
miR-21-3p, miR-125b-5p, miR-181d-5p				
miR-940	Hypoxic OC cells	Macrophages	Induce polarization of M2-phenotype macrophages	(Chen et al., 2017; Chen et al., 2018)
miR-29a-3p				
miR-21-5p	TAMs	CD4^+^ T cells	Suppress STAT3 and result in the imbalance of Treg/Th17 cells	Zhou et al. (2018)
miR-141-3p	OC cells	HUVECs	Promotes endothelial cells migration and angiogenesis	Masoumi-Dehghi et al. (2020)
miR-205	OC cells	HUVECs	Increases microvessel density	He et al. (2019)

OC, ovarian cancer; CAFs, cancer-associated fibroblasts; TAMs, tumor-associated macrophages; HUVECs, human umbilical vein endothelial cells.

OC is different from other neoplasms in that it prefers to invade the abdominal cavity through ascites. This strategy makes OC particularly adept at involving the omentum and various abdominal organs (Lheureux et al., 2019). Exosomal miR-21 derived from OC ascites regulates programmed cell death four to facilitate oncogenic transformation in distant target cells without direct colonization by tumor cells (Cappellesso et al., 2014). Ascites-derived exosomal miR-6780b-5p promotes the epithelial–mesenchymal transition (EMT) of OC cells, a key step in the invasion and metastasis (Cai et al., 2021). In addition, similar to epithelial cells that acquire fibroblast-like motility and phenotype in EMT, “mesothelial–mesenchymal transition” (MMT) refers to PMCs being converted into cancer-associated fibroblasts (CAFs) with a mesenchymal phenotype, which leads to tissue fibrosis and peritoneal adhesions (Si et al., 2019; Yang Z. G. et al., 2021). The lncRNA SPOCD1-AS is derived from the sEVs of OC. It remodels PMCs to induce the MMT phenotype by interacting with Ras-GTPase-activating protein-binding protein 1 (G3BP1), which promotes peritoneal implantation and dissemination of OC cells (Wang C. et al., 2021). circWHSC1 shows high expression in OC cells. It acts on the peritoneal mesothelium in the form of exosomes to induce tumor metastasis through sponging miR-145 and miR-1182 (Zong et al., 2019). Exosomal circPUM1 promotes peritoneal metastasis of OC via increasing NF-κB and MMP-2 expression in PMCs (Guan et al., 2019). In addition, OC cells-secreted exosomal miR-630 can induce normal fibroblasts transformation into CAFs by remodeling the extracellular matrix, and trigger tumor progression via targeting Kruppel-like factor 6 (Cui et al., 2021).

Tumor-associated macrophages (TAMs), regulatory T cells (Tregs), and T helper 17 (Th17) cells in the immune microenvironment are also crucial for OC progression (Gordon and Martinez, 2010). M2-phenotype TAMs remodel the pre-metastatic TME by suppressing the adaptive immune response (Li and Tang, 2020; Zhao et al., 2022). The Treg/Th17 ratio in the peritoneum of patients with metastatic OC is significantly higher than that in benign ovarian tumors and a benign peritoneum (Zhou et al., 2018). Moreover, Szajnik et al. (2010) have shown that tumor-derived exosomes in OC patients enhance the anti-apoptotic ability of Tregs, which may promote immune evasion of tumor cells. Hypoxia-induced overexpression of miR-21-3p, miR-125b-5p (Chen et al., 2018), and miR-940 (Chen et al., 2017) in OC cells-derived exosomes stimulate the polarization of M2-phenotype macrophages. Interestingly, Hu et al. (2017)have observed that exosomes secreted from tumor necrosis factor-like weak inducer of apoptosis (TWEAK)-stimulated macrophages can be internalized by OC cells and block metastasis thanks to miR-7 inhibiting EGFR/AKT/ERK pathway.

Vascular endothelial growth factor receptor 1 (VEGFR1)+ hematopoietic progenitor cells have initiated angiogenesis before tumor colonization to a metastatic site (Ribatti et al., 2007). This antecedent angiogenic microenvironment enables the pre-metastatic niche to meet the nutritional requirements for subsequent rapid metastasis and growth of tumor cells (Azarin et al., 2015). Furthermore, OC-derived exosomes advance angiogenesis by triggering migration of vascular endothelial cells, which contributes to tumor cells colonization into the pre-metastasis niche (Tang et al., 2018). Zhang X. et al. (2021) have found that prokineticin receptor-1(PKR1)+ exosomes also facilitate migration of vascular endothelial cells and tube formation by STAT3 phosphorylation. Masoumi-Dehghi et al. (2020) have suggested that epithelial ovarian cancer cells-derived sEVs can activate the intracellular reactive oxygen species (ROS)-dependent NF-κB signaling of endothelial cells. sEVs-miR-141-3p inhibits the suppressors of cytokine signaling-5 (SOCS-5) expression, thereby leading to up-regulation of VEGFR-2. Another study has found that OC cells-secreted exosomal miR-205 promotes metastatic progression and microvessel density by regulating the PTEN-AKT pathway (He et al., 2019).

## sEVs-ncRNAs in Drug Resistance of OC

A platinum agent combined with paclitaxel is the first-line chemotherapy regimen for OC patients (Jelovac and Armstrong, 2011). Platinum binds with double-stranded DNA to form platinum–DNA adducts, which interfere with DNA replication and RNA transcription and, eventually, initiate apoptosis. Paclitaxel is an antimitotic agent. It binds specifically to intracellular *β*-tubulin, and induces tubulin polymerization to hyper-stable microtubules, thereby suppressing mitosis to trigger apoptosis. PARPi kills OC cells in HRD status through a “synthetic lethal” effect (Kurian et al., 2021). However, multidrug resistance during chemotherapy has become a major obstacle in OC treatment (Agarwal and Kaye, 2003; Patch et al., 2015). The acquisition of drug resistance involves many factors: defects in DNA damage repair mechanisms; loss of BRCA1 promoter methylation; multiple independent reversions of gBRCA mutations; breakage of tumor suppressor genes; recurrent promoter fusion related to overexpression of multidrug-resistant protein-1 (MDR1) (Sarkadi et al., 2006; Christie and Bowtell, 2017; Norouzi-Barough et al., 2018; McMullen et al., 2020). sEVs have been shown to be important mediators for the development of resistance to anticancer drugs (Challagundla et al., 2015; Yang Q. et al., 2021). Sousa et al. (2015) have revealed that sEVs derived from tumor drug-resistant cells can confer resistant phenotypes to drug-sensitive cells via transferring miRNAs, lncRNAs, and drug-efflux pumps. In addition to tumor cells, tumor stromal cells-derived sEVs also contribute to transmitting the resistant phenotype (Zheng et al., 2017; Jia et al., 2021).

Au Yeung et al. (2016)have identified a new pathway causing paclitaxel resistance in OC cells. Exosomes secreted from cancer-associated adipose cells or CAFs transfer miR-21 into cancer cells, thereby increasing the apoptotic threshold of paclitaxel treated OC cells via down-regulating expression of apoptotic protease-activating factor-1 (APAF1). Kanlikilicer et al. (2018) have discovered that the massive release of exosomal miR-1246 from OC cells is absorbed by M2-phenotype macrophages in TME. MiR-1246 inhibitor treatment reduces expression of P-glycoprotein protein, thereby enhancing paclitaxel sensitivity of OC cells. The endogenous exosomal miR-433 inhibits the apoptosis of OC cells via down-regulating expression of phosphorylated retinoblastoma (p-Rb) and cyclin-dependent kinase-6 (CDK6), so as to induce paclitaxel resistance (Weiner-Gorzel et al., 2015). Pink et al. (2015) have found that exosomes from cisplatin-resistant OC cells can raise resistance in sensitive cells. Their data suggest that the passenger strand, miR-21-3p, directly facilitates cisplatin resistance of OC cells via targeting neuron navigator-3 (NAV3). Similarly, lncRNA urothelial carcinoma-associated-1 (UCA1) is upregulated in serum exosomes from OC patients with cisplatin-resistant. The results have shown that lncRNA UCA1 promotes cisplatin resistance of OC cells through the miR-143/FOS-like antigen 2 (FOSL2) pathway, which may be a novel target of OC therapy (Li et al., 2019). Interestingly, exosomal miR-30a-5p enhances apoptosis and sensitivity to cisplatin by regulating SRY- (SOX9) expression in OC cells (Liu et al., 2020). Feng Y. et al. (2019) have analyzed the differential expression profile of miRNAs between drug-resistant exosomes and primitive OC cells through the Gene Expression Omnibus database. They have suggested that miR-922, miR-574-3p, and miR-30a-5p may induce drug resistance by modulating a Cullin 2-mediated hypoxia-inducible factor-1 (HIF-1) pathway, whereas miR-183-5p promotes drug resistance via regulating expression of methyl-CpG-binding protein 2 (MECP2). A recent study (Zhang et al., 2021c) has indicated that sEVs-derived miRNAs can regulate oxaliplatin-induced peripheral neuropathy and chemotherapy efficacy in OC treatment. Table 2 summarizes the gene targets and mechanisms of sEVs-ncRNAs associated with resistance to OC chemotherapy.

**TABLE 2:** Summary of sEVs-ncRNAs involved in OC chemoresistance.

sEVs-ncRNAs		Targets	Drugs	Roles/Mechanisms
miR-21	APAF1	Paclitaxel	Suppresses apoptosis and confers chemoresistance on OC cells	Au Yeung et al. (2016)
miR-1246	Caveolin-1	Paclitaxel	Regulates the Caveolin-1/P-gp/M2-phenotype macrophage axis to induce drug resistance	Kanlikilicer et al. (2018)
miR-433	CDK6	Paclitaxel	Drives senescence of OC cells to induce chemoresistance	Weiner-Gorzel et al. (2015)
miR-21-3p	NAV3	Cisplatin	Contributes to cisplatin resistance via down-regulation of NAV3	Pink et al. (2015)
lncRNA UCA1	FOSL2	Cisplatin	Regulates the miR-143/FOSL2 signaling to confer chemoresistance on OC cells	Li et al. (2019)
lncRNA NEAT1	SOX3	Cisplatin	Sponges miR-491-5p to inhibit OC apoptosis	Jia et al. (2021)
miR-429	CASR	Cisplatin	Enhances proliferation of OC cells by targeting CASR/STAT3 pathway	Li et al. (2021b)
miR-21-5p	PDHA1	Cisplatin	Promotes glycolysis and OC cells viability	Zhuang et al. (2021)
miR-21-3p				
miR-21-5p				
miR-891-5p	MYC, CNBP	Carboplatin	Activate glycolysis and increase expression of DNA repair proteins	Alharbi et al. (2020)
miR-223	PTEN	Cisplatin	Promotes drug resistance and malignant phenotypes of OC cells via the PTEN-PI3K/AKT pathway	Zhu et al. (2019)
miR-4315	Bim	Anti-PD1	Induces apoptosis-resistance phenomenon via down-regulation of Bim	Guyon et al. (2020)
miR-30a-5p	SOX9	Cisplatin	Enhances OC apoptosis and cellular sensitivity to cisplatin	Liu et al. (2020)
miR-146a	LAMC2	Docetaxel	Inhibits OC cells growth and increases chemosensitivity by targeting the LAMC2-mediated PI3K/Akt axis	Qiu et al. (2020)

OC, ovarian cancer; P-gp, P-glycoprotein protein; PD-1, programmed cell death protein 1 .

## sEVs-ncRNAs as Diagnostic and Prognostic Biomarkers of OC

The prospect of significantly improved OC survival is based on an early diagnosis. Serum CA125 and human epididymis protein (HE4) are the most widely used biomarkers in the clinical diagnosis of OC. However, because of their low sensitivity and specificity, CA125 and HE4 are usually employed to monitor the progression or recurrence of OC rather than early detection. A randomized study has illustrated that the false-positive results of serum CA125 combined with transvaginal ultrasound screening lead to unnecessary surgical procedures and serious complications (Partridge et al., 2013). It has been demonstrated that the ncRNA composition in sEVs is a “snapshot” of parental cells status, in which cargoes with specific changes could be used as key biomarkers of diseases (Royo and Falcon-Perez, 2012). sEVs-derived ncRNAs as noninvasive biomarkers in liquid biopsies have three main advantages. First, they are quite stable (not degraded by RNase) (Möller and Lobb, 2020). Second, the vesicle surface displays the antigenic markers of original cells, which allows enrichment of vesicles from specific tissue sources (e.g., capture of OC-derived sEVs in plasma using EpCAM antibody) (Nagrath et al., 2007). Third, the content of sEV-ncRNAs is related to tumor stage and treatment outcome (Yokoi et al., 2018).

Taylor and Gercel-Taylor (2008) have separated tumor cells-derived exosomes from the plasma of OC patients using an improved immunoaffinity magnetic bead-sorting system with anti-EpCAM. Their microarray data has suggested that the “signature” of circulating tumor-derived exosomal miRNAs mirror and exhibit a strong correlation with the profile of tumor tissue-derived miRNAs in the same OC patients (exhibiting correlations from 0.71 to 0.90). Hence, circulating tumor-derived exosomal miRNAs can be used as candidate biomarkers for OC diagnosis. Another study has illustrated that there are a large number of abnormally expressed miRNAs (miR-106a-5p, let-7d-5p, miR-93-5p, miR-122-5p, miR-185-5p, miR-99b-5p) in plasma and exosomes of OC patients compared with healthy controls (Zhang et al., 2019). Moreover, the concentration of serum exosomal miR-373, miR-200a, miR-200b, and miR-200c in OC patients have been found to be significantly higher than those in healthy women and to be associated with tumor stage (Meng et al., 2016). Interestingly, expression of exosomal miR-34a in the serum of early-stage OC patients is significantly higher than that in advanced OC patients, and is negatively correlated with the risk of lymph-node metastasis and OC recurrence (Maeda et al., 2020).

sEVs-derived miRNAs can also be employed to distinguish the different histological types of OC. Kobayashi et al. (2018) have found a significantly increased expression of miR-1290 in the serum of HGSOC patients, which can distinguish HGSOC from patients with other histological types of OC. Importantly, miRNA expression of circulating exosomes does not always parallel that of the original tumor tissue. Kim et al. (2019) have discovered that miR-145 (identified as a down-regulated biomarker in OC tissue) is highly expressed in the serum exosomes of OC patients. Moreover, low expression of exosomal miR-484 in serum has been associated significantly with aggressive clinical variables and shorter overall survival and progression-free survival (Zhang W. et al., 2020). Wang et al. (2020) have detected the exosomal circRNAs expression profile in serum between OC patients and healthy subjects. Serum exosomal circ-0001068 expression in OC patients is up-regulated significantly compared with that in healthy subjects. OC cells-derived circ-0001068 is delivered into T cells as a comepting endogenous RNA of miR-28-5p, resulting in increased expression of programmed cell death protein 1 (PD1) and T-cell exhaustion. They have suggested that circ-0001068 may be a novel noninvasive diagnostic biomarker and therapeutic target for OC.

## Engineering sEVs Targeted Delivery of ncRNAs in OC Therapy

**FIGURE 3:**
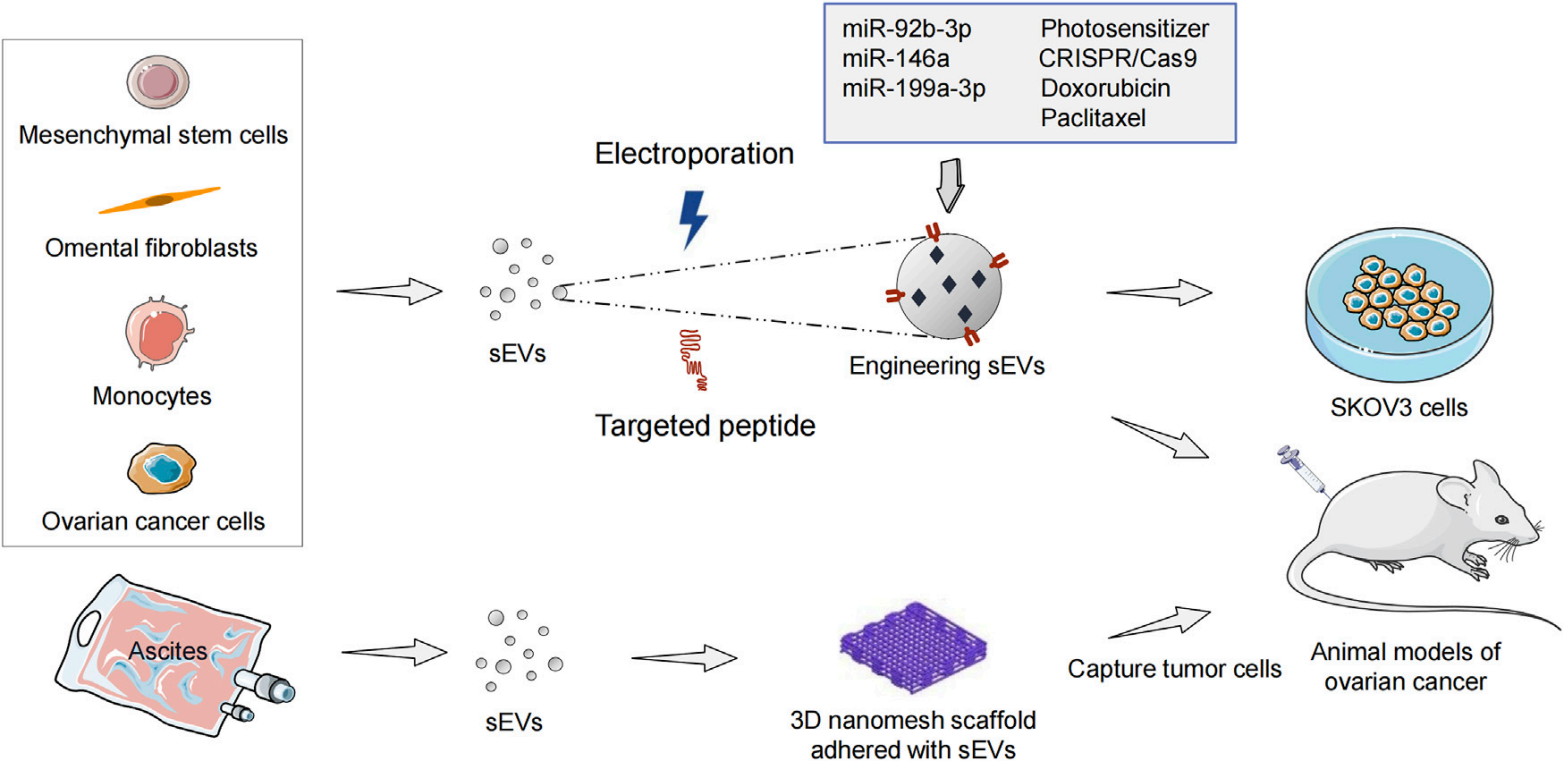
Engineering sEVs targeted delivery of ncRNAs and anticancer drugs in ovarian cancer treatment. sEVs, small extracellular vesicles; ncRNA, non-coding RNA.

Extensive efforts are being devoted to developing therapeutic strategies that can effectively bypass the physical and biological barriers within the peritoneum in metastatic OC patients. A new direction of OC therapy is to develop various targeted delivery systems based on drug conjugates and nanomedicines (Wang Z. et al., 2021). As biogenic and bioactive nanovectors for cancer therapy, sEVs include the following advantages. First, therapeutic biomaterials can be loaded into sEVs. Second, they can be absorbed by recipient cells, thereby altering cellular processes. Third, sEVs have high histocompatibility and can reduce immune clearance in drug delivery (Kamerkar et al., 2017; Al-Dossary et al., 2021). As drug-delivery vesicles, sEVs can penetrate through anatomical barriers and have been shown to deliver miRNAs (Jc Bose et al., 2018), siRNAs (Kamerkar et al., 2017), CRISPR/Cas9 (Yao et al., 2021), and chemotherapeutic drugs (Qiu et al., 2019) safely and effectively in animal models (Figure 3).

Pinto et al. (2021) have found sEVs from mesenchymal stem cells (MSCs) to be immune-active photosensitizers (PS) vectors that can be employed to treat OC patients with peritoneal metastasis. Intraperitoneal injection of MSCs-derived sEVs-PS in model mice significantly enhances tumoral targeting compared with liposomal (Foslip) and the free drug. The mean ratio of PS in tumors/organs is 40 in the sEVs-PS group, 5.5 in the Foslip group, and 1.5 in the free PS group. sEVs-PS mediated photodynamic therapy promotes 55% of tumoral nodules necrosis (sEVs-PS versus Foslip [*p* < 0.0001]) and enhances CD8^+^ effector T cells infiltration to significantly prolong mice survival. Fuente et al. (2015) have embedded exosomes purified from ascites of OC patients into 3D scaffolds (metastatic trap [M-Trap]) to capture tumor cells disseminated in the peritoneal cavity, thereby interfering with the natural process of peritoneal metastasis. After the M-Trap device is applied to the murine model of OC with peritoneal metastasis, the mean survival increases from 117.5 to 309.4 days (*p* < 0.001). Overall, these findings support the importance of sEVs in the treatment of OC with peritoneal metastasis.

Numerous studies indicate that inhibiting the function of overexpressed oncogenic miRNA (oncomiR) is a potent anticancer therapeutic strategy. MiR-21 is a robust oncomiR in most cancers (Abels et al., 2019) as well as OC (Cappellesso et al., 2014). Antisense miR-21 therapy can enhance apoptosis and chemosensitivity in OC cells (Chan et al., 2014; Raniolo et al., 2021). Targeted therapy of engineering sEVs based on antisense miR-21 has achieved good efficacy in animal models of breast cancer (Jc Bose et al., 2018), and colorectal cancer (Liang et al., 2020). Wang J. et al. (2021) have investigated the potential of peptide-engineered exosomes overexpressing miR-92b-3p in anti-angiogenic treatment of OC. Their data has shown that DSPE-PEG2K-RGD modified exosomes packaged with miR-92b-3p can produce combined antitumor and antiangiogenic effects with Apatinib in intraperitoneal tumorigenesis model of nude mice. Qiu et al. (2020) have found that human umbilical cord MSCs-derived exosomes carrying miR-146a increase the sensitivity of OC cells to docetaxel and taxane by targeting the laminin gamma-2 (LAMC2)-mediated PI3K/Akt axis. In another xenograft study, miR199a-3p encapsulated in omental fibroblasts-derived exosomes significantly inhibits the peritoneal dissemination of OC cells in a mice model (Kobayashi et al., 2020).

CRISPR/Cas9 is a therapeutic genome editing technology, which has great application prospects in OC. Tumor-derived exosomes carrying CRISPR/Cas9 via electroporation disrupt PARP-1 in SKOV3 cells, resulting in apoptosis and enhancing chemosensitivity to cisplatin (Kim et al., 2017). As a carrier with low immunogenicity, exosomes achieve efficient CRISPR/cas9-mediated genome editing *in vivo*. However, the biggest challenges for sEVs-based clinical treatment are large-scale production, isolation, purification, and modification. Pisano et al. (2020) have developed an exosome production platform based on monocytes. Doxorubicin (DOX) is injected into exosomes as a model drug to treat SKOV3 cells. Monocytes-derived immune exosomes have shown higher production yields (2.5-fold increase), encapsulation efficiency, and drug release rate than natural exosomes. In addition, Hadla et al. (2016) have demonstrated that exosomes not only increase the therapeutic index of DOX in OC mouse models, but also avoid the cardiotoxic side effects caused by drug accumulation in myocardial endothelial cells. Paclitaxel-loaded MSCs-derived exosomes (MSCs-exos) significantly reduce the number of SKOV3 cells through apoptotic and necrotic disintegration (Melzer et al., 2019). Compared with the equivalent cytotoxic obtained via paclitaxel *in vitro*, the concentration of paclitaxel in MSCs-exos is reduced 7.6-fold, which indicates a higher specificity and tumor targeting of MSCs-exos.

The sEVs delivery system loaded with siRNAs or anticancer drugs is a promising approach for the treatment of OC patients with peritoneal metastasis. However, the efficacious drugs to eliminate harmful sEVs in OC patients are not available. Five efficacious exosome inhibitors have been identified through high-throughput drug screening: ketoconazole, triadimenol, tipifarnib, climbazole, and neticonazole. Their efficacy *in vivo* needs further validation (Datta et al., 2018). Furthermore, it has been found that an inhibitor of the ceramide-biosynthesis regulator, GW4869, can damage the exosomal secretion of 293T cells (Essandoh et al., 2015). Those findings indicate that the application of sEV inhibitors in OC treatment is worthy of further exploration.

## Conclusion and Outlook

Many experimental studies have revealed the importance of sEVs-derived ncRNAs in OC progression and resistance to platinum-based chemotherapy. Hence, sEVs-ncRNAs from biological fluids (e.g., plasma or ascites) can indicate tumor status and an adaptative pathologic response to drug exposure in OC patients. Compared with the difficulty of obtaining tumor tissue, detection of sEVs-ncRNAs in plasma can aid the early diagnosis of OC, monitor tumor progression, and assess drug resistance in real-time. Furthermore, sEVs-ncRNAs, as quasi-fragments derived from tumor cells, allow for the integration of multiple molecular biomarkers to establish the diagnostic model. Studies have indicated that the diagnostic performance of the sEVs miRNAs model appears to be equivalent to or even better than the circulating miRNAs model. However, most currently available OC biomarkers based on sEVs-ncRNAs require further prospective validation. Previous studies are not directly comparable in terms of study design, investigation population, methods of sEVs isolation, and biopsy materials. Therefore, reproducible results must be achieved in large-scale studies based on patient stratification and standardized isolation methods for sEVs-ncRNAs.

sEVs that have been optimized naturally over a long period can provide unique advantages over synthetic nanoparticles. Engineering sEVs-based nanotechnology has made great strides in OC treatment, but this has not translated to longer survival for OC patients. Thus, there is a need to use sEVs-RNAi or immunotherapy (or a combination of the two options) to target OC tumors (especially in the peritoneal cavity) rather than relying on a passive therapeutic mechanism. Additionally, to become clinically usable therapeutic vectors, sEVs must face the challenges of highly controlled purification and separation, industrial-scale production, loading efficiency, and long-term efficacy. On the other hand, achieving translational application of sEVs requires direct quantitative comparisons with clinical therapy systems of OC, such as liposomal-based drug therapy. More importantly, the natural content contained in sEVs will be jointly delivered to target cells and may play a biological role. Their harmful side-effects remain to be determined, and the safety and efficacy of sEVs-based drug delivery *in vivo* need further evaluation. With the development and optimization of research methods, sEVs-ncRNAs are expected to promote the formulation of novel diagnosis and treatment strategies for OC.
